# Pathological roles of MRP14 in anemia and splenomegaly during experimental visceral leishmaniasis

**DOI:** 10.1371/journal.pntd.0008020

**Published:** 2020-01-21

**Authors:** Kanna Ishizuka, Wataru Fujii, Natsuho Azuma, Haruka Mizobuchi, Ayako Morimoto, Chizu Sanjoba, Yoshitsugu Matsumoto, Yasuyuki Goto

**Affiliations:** 1 Laboratory of Molecular Immunology, Department of Animal Resource Sciences, Graduate School of Agricultural and Life Sciences, The University of Tokyo, Tokyo, Japan; 2 Laboratory of Applied Genetics, Department of Animal Resource Sciences, Graduate School of Agricultural and Life Sciences, The University of Tokyo, Tokyo, Japan; Ohio State University, UNITED STATES

## Abstract

Myeloid-related protein 14 (MRP14) belongs to the S100 calcium-binding protein family and is expressed in neutrophils and inflammatory macrophages. Increase in the number of MRP14^+^ cells or serum level of MRP14 is associated with various diseases such as autoimmune diseases and infectious diseases, suggesting the involvement of the molecule in pathogenesis of those diseases. In this study, to examine the pathological involvement of MRP14 during cutaneous and visceral leishmaniasis, wild-type (WT) and MRP14 knockout (MRP14KO) mice were infected with *Leishmania major* and *L*. *donovani*. Increase in the number of MRP14^+^ cells at the infection sites in wild-type mice was commonly found in the skin during *L*. *major* infection as well as the spleen and liver during *L*. *donovani* infection. In contrast, the influence of MRP14 to the pathology seemed different between the two infections. MRP14 depletion exacerbated the lesion development and ulcer formation in *L*. *major* infection. On the other hand, the depletion improved anemia and splenomegaly but not hepatomegaly at 24 weeks of *L*. *donovani* infection. These results suggest that, distinct from its protective role in CL, MRP14 is involved in exacerbation of some symptoms during VL.

## Author summary

Inflammatory responses are crucial in limiting pathogen infections, whereas they often have detrimental effects to the hosts during infectious diseases. Thus, exploring molecules involved in inflammatory responses and targeting those molecules may contribute to development of novel interventions for symptom management during infectious diseases. Although an inflammation-related protein, MRP14, is associated with a wide range of inflammatory diseases, the functions remain elusive. Therefore, we explored the roles of MRP14 in a protozoan disease called leishmaniasis because its pathology is known to be immune-mediated. Interestingly, in two distinct forms of leishmaniases, i.e., cutaneous leishmaniasis caused by *Leishmania major* and visceral leishmaniasis caused by *L*. *donovani*, the molecule showed distinct roles in protection/pathology; although the molecule was protective against *L*. *major* infection, depletion of the molecule resulted in easing the pathology during *L*. *donovani* infection. These may prove the complexity of MRP14, but at the same time support understanding of the mechanisms behind the complexity.

## Introduction

Myeloid-related protein (MRP) 14, also known as S100A9, belongs to the S100 calcium-binding protein family and can form the heterodimer with MRP8, which is known as S100A8 [1,2]. S100 protein family proteins contain two Ca^2+^-binding regions known as EF-hands and play a role in cell differentiation, cell cycle progression, regulation of kinase activity, and cytoskeletal-membrane interactions when Ca2^+^ bind [3]. The expression of MRP14 and MRP8 is specific for myeloid cells such as granulocytes, monocytes and macrophages in inflamed tissue [4]. They are abundant cytoplasmic proteins of neutrophils and monocytes [1,5], and also known as markers of inflammatory macrophages. MRP14 expression is abundant in immature monocytes and is lost as the cells terminally differentiate into tissue macrophages, so MRP14 can be associated with monocytic differentiation [6].

MRP14 has been characterized as an inflammation-related protein [7–9]. Extracellular MRP14 and MRP8 are known to function as damage-associated molecular patterns (DAMP), which are endogenous molecules or alarmins released after cell activation or necrotic cells and are secreted by the inflammatory cells when activated [10]. Neither MRP14 nor MRP8 has a signal sequence for secretion via classical ER/Golgi route, but it is demonstrated that these proteins are secreted after activation of protein kinase C via tubulin-dependent pathway [10]. Extracellular MRP14 and MRP8 bind Toll-like receptor (TLR) 2, TLR4 and receptor for advanced glycation endproducts (RAGE) and induce cell recruitment and cell activation [11–14]. It is also reported that MRP14 promotes inflammatory process in infection and autoimmunity via TLR4 [9,12,15], and cell growth in cancer and cell migration via RAGE [16,17]. Actually, MRP8 and MRP14 in serum are elevated in various diseases [18–20]. In inflammatory diseases such as rheumatoid arthritis, psoriatic arthritis, and coronary syndromes, the accumulation of cells expressing MRP14 or MRP8 is observed at inflammatory sites [18,21–23]. Therefore, it is considered that MRP14 plays a critical role in the pathogenesis in these diseases. In malaria, we reported that macrophages expressing MRP14 accumulated in the spleen and liver of BALB/c mice and MRP14 level in the plasma was also elevated during *Plasmodium berghei* ANKA infection [24]. In addition, the administration of recombinant MRP14 exacerbated hepatic injury and promoted the up-regulation of pro-inflammatory molecules in the liver [11]. These reports suggest MRP14 is one of the key molecules for pathogenesis of malaria. In the present study, pathological involvement of MRP14 in leishmaniasis, which is caused by parasite infection as well as malaria, was examined.

The leishmaniasis is caused by protozoan parasites of the genus *Leishmania*. Human leishmaniasis is typically classified into three types, cutaneous leishmaniasis (CL), mucocutaneous leishmaniasis and visceral leishmaniasis (VL). *Leishmania major* is one of the causative agents for CL, which is characterized by clinical manifestations such as ulcers on the skin and permanent scars after the ulcers heal. VL, also known as kala-azar, is caused by infection of *Leishmania* species including *L*. *donovani* and *L*. *infantum* [25,26] and is characterized by clinical manifestations such as fever, substantial weight loss, hepatosplenomegaly, and anemia. During CL and VL, macrophages are the host cells of *Leishmania* parasites in the mammalian hosts, and the parasites proliferate within macrophages in the skin lesion and lymph node during CL and in the spleen, liver, and bone marrow during VL.

During experimental CL, MRP14^+^ cells are predominant cells accumulated in the skin lesion during *L*. *major* infection in BALB/c mice [27]. A study using MRP14 knockout (MRP14KO) C57BL/6 mice showed a protective role of MRP14 in *L*. *major* infection to footpad [28]. Such the protective role has been confirmed also in BALB/c mice as injection of recombinant MRP14 into the lesion induced reduction in parasite burden and suppression of lesion development [28]. In contrast, the role of MRP14 in *L*. *donovani* infection has not been studied yet. Because the symptoms are very different between CL and VL, the role of MRP14 in pathology of VL may be different from that in CL. Therefore, by utilizing MRP14KO BALB/c mice, we addressed the influence of MRP14 on pathology during *L*. *donovani* infection and compared with *L*. *major* infection. The results revealed that the molecule can exacerbate splenomegaly and anemia during *L*. *donovani* infection whereas it can contribute to control of *L*. *major* infection. By comparing the influence of MRP14 during *L*. *major* and *L*. *donovani* infection, it was revealed that how MRP14 involves in pathogenesis is variable.

## Materials and methods

### Animals

BALB/cA mice were purchased from Japan Clea, Tokyo, Japan, and were maintained under specific pathogen-free conditions. MRP14KO BALB/cA mice were generated by offset-nicking method of CRISPR/Cas system according to previous report [29,30]. Mice were bred in the animal facility at the Graduate School of Agricultural and Life Sciences, The University of Tokyo. The mice were used for experiments at the age of 6–9 weeks. The animal experiments were reviewed and approved by an institutional animal research committee (Approval No. P16-285) and an institutional committee on genetically modified organisms (Approval No. 830–2630) at the Graduate School of Agricultural and Life Sciences, The University of Tokyo. Animal health and well-being was assessed in accordance with the Guidelines for Proper Conduct of Animal Experiments (the Science Council of Japan) and the National Institutes of Health guidelines for the use of experimental animals.

### Parasites

*L*. *major* promastigotes (MHOM/IL/80/Friedlin; provided by Dr. Steven Reed, Infectious Diseases Research Institute) were cultured in medium TC199 supplemented with 20% HI-FBS and 25 mM HEPES buffer at 25°C. *L*. *donovani* promastigotes (MHOM/NP/03/D10; a gift from the National BioResource Project at Nagasaki University [31]) were cultured in medium TC199 (Nissui Pharmaceutical, Tokyo, Japan) supplemented with 10% heat-inactivated fetal bovine serum (HI-FBS; Thermo Scientific, Waltham, USA) and 25 mM HEPES buffer (MP Biomedicals, France) at 25°C.

### Animal infection

Preparation of metacyclic promastigotes was performed using peanut agglutinin as previously described [32]. Mice were infected with 1 × 10^4^ *L*. *major* metacyclic promastigotes by intradermal injection into the left ear [33]. The longer axis of the lesion and that of the ulcer were measured with a digital caliper every week [34]. These mice were sacrificed at 10 weeks after infection or earlier if severe damage to the auricle was evident. The number of parasites in lymph nodes at sacrifice was determined by limiting dilutions [35]. The tissues were homogenized using glass homogenizer in 1 ml of TC199 medium containing 20% HI-FBS, 25 mM HEPES buffer, penicillin (100 U/ml) and streptomycin (100 μg/ml) (Invitrogen, CA, US). Two-fold serial dilutions of the homogenates were made in 96 well plates up to 1:2^23^. After 14 days of incubation at 25°C, the plates were examined for the presence of promastigotes. The parasite number in each sample was calculated by defining the last positive well as containing one amastigote at the beginning. Two independent *L*. *major* infections were performed.

*L*. *donovani* promastigotes in stationary phase were washed with phosphate-buffered saline (PBS: Nissui Pharm) by centrifugation at 1,600 ×*g* for 10 min and were resuspended with PBS at the concentration of 1 × 10^8^ cells/ml. Mice were infected with 1 × 10^7^ *L*. *donovani* promastigotes by intravenous injection into the tail vein. Twenty-four weeks after infection, the mice were sacrificed by cervical dislocation to collect the spleen and liver. Stamp smears of the spleen and liver were fixed for 5 min in methanol and stained for 25 min with 5% Giemsa solution (Merck KGaA, Parmstadt, Germany). Amastigotes were counted by microscopic observation of the stained smear at 1,000× magnification, and Leishman-Donovan Units (LDU) were enumerated as the number of amastigotes per 1,000 host nuclei times the tissue weight in grams as performed in a previous study [36]. Three independent *L*. *donovani* infections were performed.

### Immunohistochemical analyses

Immunohistochemical staining was performed as previously described [24]. Briefly, for immunohistochemical staining, paraffin-embedded tissues, sectioned at 4 μm thickness, were dewaxed and boiled in Tris-EDTA buffer (10 mM Tris Base, 1 mM EDTA-2Na, 0.05% Tween 20, pH 9.0) for 20 minutes. After blocking, anti-MRP8 and anti-MRP14 antibody (Santa Cruz Biotechnology, Texas, USA) was applied to the serial sections of tissues. After washing with PBS, sections were incubated with biotinylated anti-goat IgG (Nichirei Bioscience, Tokyo, Japan). Finally, enzymatic color development was performed by using 4-[(4-amino-m-tolyl) (4-imino-3-methylcyclohexa-2, 5-dien-1-ylidene) methyl]-o-toluidine monohydrochloride (new fuchsine, Nichirei Bioscience).

The number of MRP8^+^ cells in the immunohistochemically stained spleen were counted in 10 random microscopic fields at 200× magnification. For the spleen, the cells in the red pulp were counted.

### HE staining

The skins were collected from *L*. *major*-infected mice after 10 weeks of infection, and the tissues were fixed with 20% neutral buffered formalin and embedded in paraffin. The paraffin-embedded tissues were sectioned at 4 μm thickness. The tissues were stained with Mayer’s hematoxylin solution (WAKO, Osaka, Japan) for 1.5 min and rinsed in running tap water for 30 min. Next, the tissues were stained with eosin solution (MUTO PURE CHEMICALS CO., Ltd., Tokyo, Japan) for 2 min.

### Quantitative RT- PCR

RNA from the spleens from *L*. *donovani*-infected mice was extracted using TRIzol (Thermo Fisher Scientific) and cDNA was synthesized by reverse transcription. The spleens were homogenized with 1 ml TRIzol and φ1.0 stainless steel beads in the 2 ml tube using Micro Smash MS100R (Tomy Seiko) at 4°C and RNA was purified according to the manufacture’s protocol. The concentration of total RNA was measured by DU 730 Life Science UV/vis spectrophotometer (Beckman Coulter). A mixture of 1.25 μM oligo (dT)_16_, 0.5mM dNTPs (Thermo Fisher Scientific) and 1 μg of total RNA in a tube was incubated for 5 min at 65°C. After adding 5× first strand buffer and 10 mM DTT (Thermo Fisher Scientific), 200 U M-MLV (Thermo Fisher Scientific) was added and the tube was incubated for 50 min at 37°C and 15 min at 70°C. Real-time PCR assay was carried out using 2 μl of cDNA as the template, 10 μl of SYBR Select Master Mix (Thermo Fisher Scientific) and primers listed in S1 Table [11,37] on the ABI Prism 7000 Sequence Detection System (Thermo Fisher Scientific). Data was analyzed by 2^−ΔΔCt^ methods and normalized by GAPDH. The thermal cycling conditions for the PCR were 94°C for 10 min, followed by 40 cycles of 94°C for 15 sec and 60°C for 1 min.

### T cell assay

To examine T cell reactivity, 2 × 10^5^ cells per well of splenocytes from *L*. *major*- and *L*. *donovani-*infected WT and MRP14KO mice in RPMI-1640 medium (Sigma-Aldrich) supplemented with 10% HI-FBS, 100 U/ml of penicillin and 100 μg/ml of streptomycin were plated in a 96-well round-bottom plate (Thermo Fisher Scientific) and stimulated with 3 μg/ml of concanavalin A, 10 μg/ml of *L*. *major-* or *L*. *donovani-* derived soluble lysate antigen (LmSLA or LdSLA), or medium alone. Culture supernatants were collected after 72 hours cultivation in 5% CO_2_ at 37°C and tested the levels of interferon-γ (IFN-γ) by using Mouse IFN gamma ELISA Ready-SET-Go! Kit (eBioscience) according to manufacturer’s instruction.

### Statistical analyses

Statistical analyses were performed using GraphPad Prism 6 software (GraphPad Software, Inc., La Jolla, USA). Mann-Whitney test was used to compare the differences in the results from two independent groups. The differences in MRP8^+^ cell accumulation between WT and MRP14KO mice were analyzed by two-way ANOVA followed by Sidak’s multiple comparisons test. A difference between groups was considered as statistically significant when the *P* value was less than 0.05.

## Results

### MRP8^+^ and MRP14^+^ cells at the infection sites in WT mice during *L*. *major* and *L*. *donovani* infection

**Fig 1:**
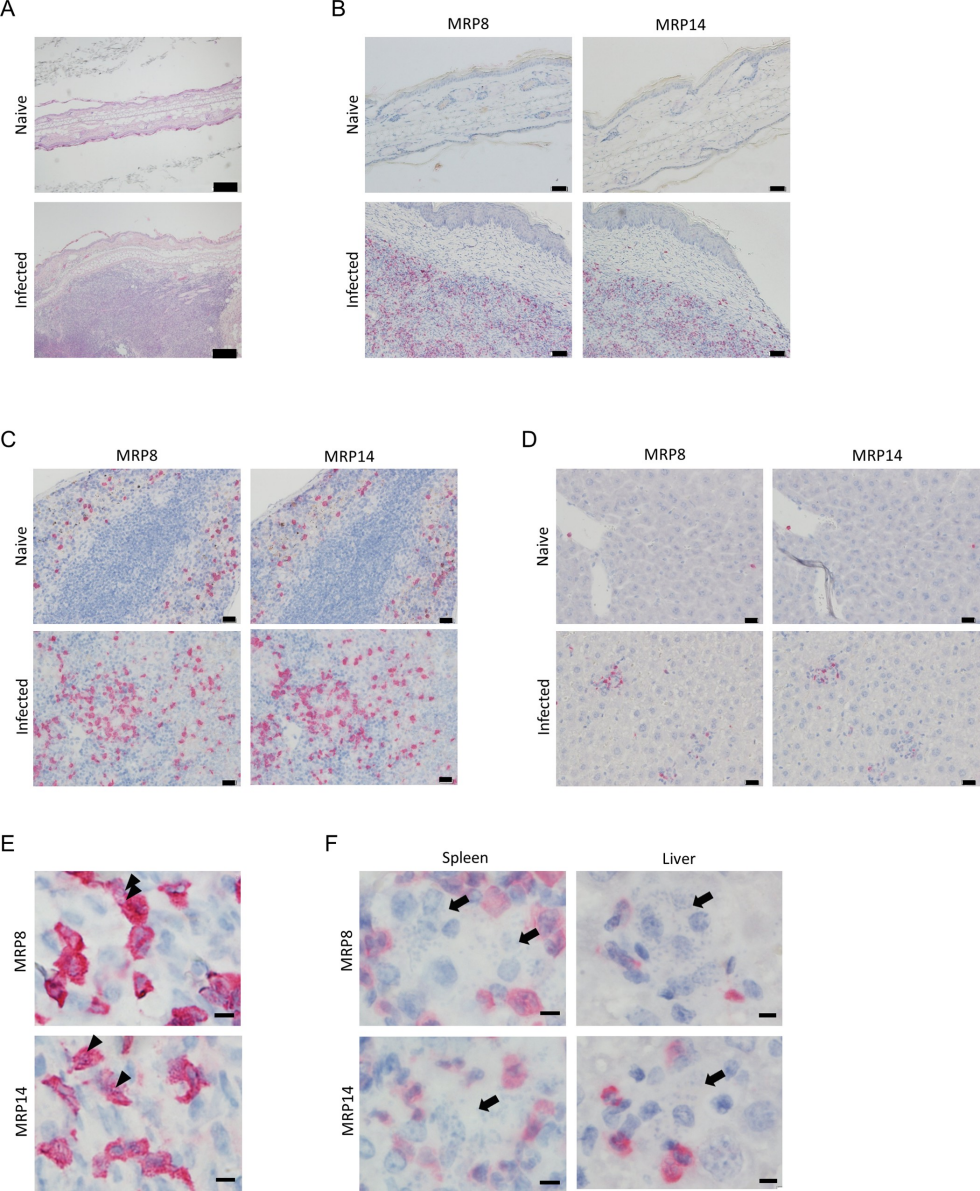
Accumulation of MRP8^+^ and MRP14^+^ cells to the infection sites during *Leishmania* infections. (A) Representative images of HE-stained skin from naïve WT mice and skin lesions from *L*. *major*-infected WT mice. Bars, 200 μm. (B) Representative images of IHC of the uninfected skin or skin lesions by anti-MRP8 or anti-MRP14 antibody. Bars, 50 μm. (C, D) Representative images of IHC of the spleen (C) or liver (D) from naïve or *L*. *donovani*-infected WT mice by anti-MRP8 or anti-MRP14 antibody. Bars, 20 μm. (E) Representative images of MRP8^+^ and MRP14^+^ cells in the skin lesion of *L*. *major*-infected WT mice at 1000× magnification. Bar, 5 μm. Arrow heads, *L*. *major* amastigotes. (F) Representative images of MRP8^+^ and MRP14^+^ cells in the spleen and liver of *L*. *donovani*-infected WT mice. Arrows, *L*. *donovani-*infected cells. Note that parasitized macrophages, which were negative for MRP8 or MRP14, were surrounded by uninfected MRP8^+^ and MRP14^+^ cells.

Microscopic observation of the HE-stained ear of *L*. *major* infected BALB/c mice at 10 weeks post infection revealed that pathological changes compared with that of the naïve mice (Fig 1A). In *L*. *major* infection, cell accumulation was found between epidermal tissues in skin lesion. To characterize the accumulated cells, the expression of MRP8 and MRP14 was examined by immunohistochemistry (IHC). Few MRP8^+^ and MRP14^+^ cells were found in skin of naïve mice, while both MRP8^+^ cells and MRP14^+^ cells were accumulated in dermal tissues of skin lesion and were predominant cell types in the lesion (Fig 1B).

To examine if accumulation of MRP8^+^ and MRP14^+^ cells is also induced during VL, IHC of the spleen and liver from *L*. *donovani* infected BALB/c mice was performed. MRP8^+^ and MRP14^+^ cells were observed in the spleen of naïve mice and their numbers increased after 24 weeks of infection (Fig 1C). MRP8^+^ and MRP14^+^ cells were found only in the red pulp of both naïve and infected mice. Although few MRP8^+^ and MRP14^+^ cells were found in the liver of naïve mice, those cells increased during *L*. *donovani* infection as a part of granuloma formations (Fig 1D).

A major difference in MRP8^+^ and MRP14^+^ cells between *L*. *major* infection and *L*. *donovani* infection was the presence of parasites in those cells. In MRP8^+^ and MRP14^+^ cells accumulated in the skin lesion of *L*. *major*-infected mice, the nuclei of parasites were often found (Fig 1E). In contrast, signal of MRP8 or MRP14 was not observed in the parasitized macrophages (Fig 1F). Especially in the liver, it was typical that granuloma formations were composed of parasitized macrophages in the middle which were negative for MRP8 or MRP14, whereas MRP8^+^ and MRP14^+^ cells were found in the surrounding area and were unparasitized (Fig 1F).

### Exacerbated lesion development in *L*. *major* infection and alleviated anemia and splenomegaly in *L*. *donovani* infection by MRP14 depletion

**Fig 2:**
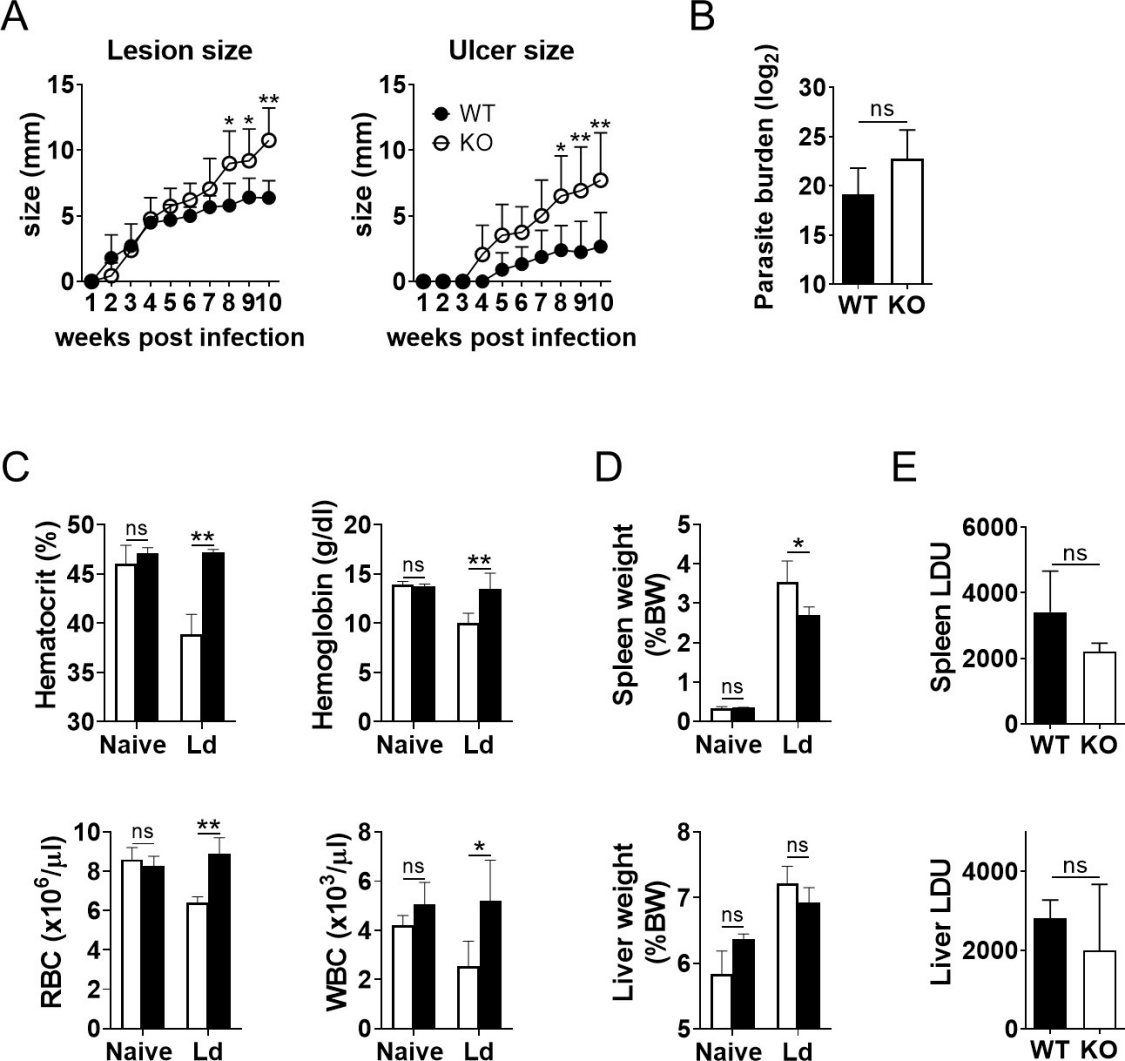
Exacerbated lesion development in *L*. *major* infection and alleviated anemia and splenomegaly in *L*. *donovani* infection by MRP14 depletion. (A) The size of the skin lesion and ulcer of the ear in *L*. *major*-infected WT (n = 5) and MRP14KO mice (n = 5) was measured every week. (B) Parasite burdens of auricular lymph nodes in *L*. *major*-infected WT and MRP14KO mice were measured by limiting dilutions. These are a representative of two independent experiments with similar results. (C, D) Hematological data (C) and organ weights (D) of *L*. *donovani*-infected WT (n = 4) and MRP14KO mice (n = 5) at 24 weeks of infection are shown. (E) Parasite burdens in the spleen and liver of *L*. *donovani*-infected WT and MRP14KO mice at 24 weeks of infection were determined by microscopy of Giemsa-stained smears and are expressed as LDU. These are a representative of three independent experiments with similar results. Means and SD of each group are shown. ns, not significant, *P < 0.05, **P < 0.01 by Mann-Whitney test.

In order to see the role of MRP14 in lesion development during *L*. *major* infection, ear lesion size and ulcer size of the infected WT and MRP14KO mice were measured every week. Although both WT and MRP14KO mice developed skin lesion and ulcer, MRP14 depletion significantly exacerbated lesion development and ulcer formation (Fig 2A). Mean length ± SD of the long diameter of the skin lesion in WT and MRP14KO mice at 10 weeks post infection were 6.4 ± 1.9 mm and 10.8 ± 2.5 mm, respectively. Mean length ± SD of the ulcer size were 2.7 ± 2.6 mm and 7.7 ± 3.6 mm, and the ulcers in MRP14KO mice were found 1 week earlier than WT mice. In contrast, mean parasite numbers in auricular lymph node were not significantly difference between the infected WT and MRP14KO mice (Fig 2B).

Next, WT and MRP14KO mice were examined for major symptoms of VL, i.e., anemia, leukopenia and hepatosplenomegaly during *L*. *donovani* infection. Before infection, hematocrit, hemoglobin levels, and red blood cell counts in peripheral blood of WT mice (48.4 ± 0.9%, 15.5 ± 1.0 g/dl, 7.8 ± 1.0 × 10^6^ cells/μl) were comparable to those of MRP14KO mice (47.9 ± 1.3%, 15.2 ± 0.9 g/dl, 7.5 ± 1.4 × 10^6^ cells/μl). In contrast, WT mice developed anemia after 24 weeks of infection represented by lower hematocrit, hemoglobin levels and red blood cells count in peripheral blood (38.9 ± 2.0%, 10.1 ± 0.9 g/dl and 6.4 ± 0.3 × 10^6^ cells/μl, respectively), whereas MRP14KO mice did not exhibit anemia showing higher hematocrit, red blood cells count and hemoglobin in peripheral blood (46.0 ± 1.9%, 13.5 ± 1.6 g/dl and 8.9 ± 0.8 × 10^6^ cells/μl, respectively) than the infected WT mice (Fig 2C). Mean ± SD of white blood cells in the infected MRP14KO mice (5,200 ± 1,651 cells/μl) were significantly different from that of WT mice (2,525 ± 1,037 /μl) (Fig 2C). Splenomegaly in the infected MRP14KO mice was milder than that in the infected WT mice whereas hepatomegaly was similarly induced in both mice. Means ± SD of spleen weights per body weight in naïve WT and MRP14KO mice were 0.26 ± 0.04% and 0.25 ± 0.02%, and those from the infected WT and MRP14KO mice were 3.54 ± 0.54% and 2.71 ± 0.20%, respectively (Fig 2C). Means ± SD of liver weights per body weight in naïve WT and MRP14KO mice were 4.83 ± 0.24% and 5.82 ± 0.08%, and those from infected WT and MRP14KO mice were 7.21 ± 0.27% and 6.92 ± 0.23% (Fig 2C). Means ± SD of LDU in the spleen of the infected WT and MRP14KO were 4,128 ± 1,209 and 2,189 ± 271, respectively (Fig 2D). Those of liver were 2951 ± 381.2 and 1991 ± 1780 (Fig 2D). There was no significant difference in either spleen or liver parasite burdens between WT and MRP14KO mice.

### MRP14-dependent accumulation of MRP8^+^ cells during infection with *L*. *major* but not *L*. *donovani*

**Fig 3:**
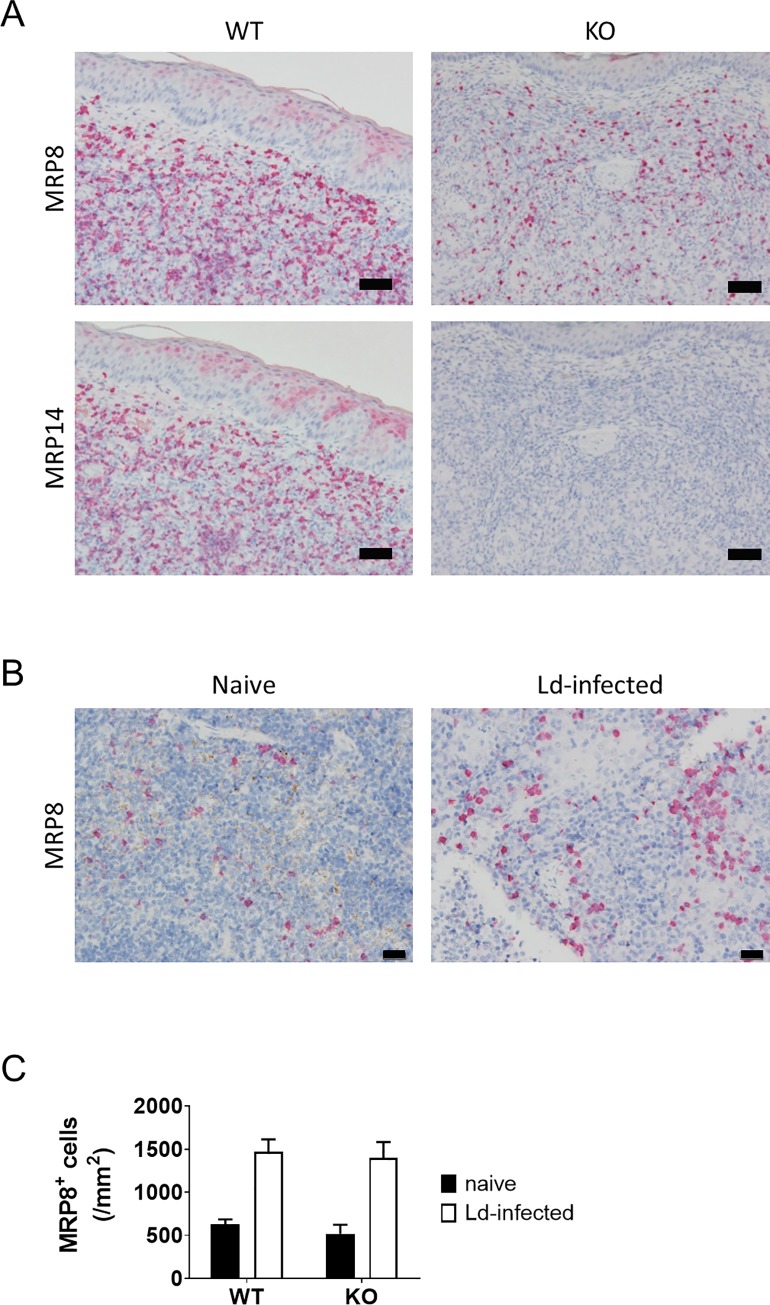
MRP14-dependent accumulation of MRP8^+^ cells during infection with *L*. *major* but not *L*. *donovani*. (A) MRP8^+^ and MRP14^+^ cells in skin lesion of the ear from *L*. *major*-infected WT and MRP14KO mice at 10 weeks of infection were analyzed by IHC. Bars, 50 μm. (B) MRP8^+^ cells in the spleen of naïve and *L*. *donovani*-infected MRP14KO mice at 24 weeks of infection were analyzed by IHC. Bars, 20 μm. (C) The number of MRP8^+^ cells in the spleen of naïve (n = 3) and *L*. *donovani*-infected mice (n = 4 or 5) at 24 weeks of infection was counted. Means and SD of WT and MRP14KO mice are shown. These are a representative of two or three independent experiments with similar results.

To investigate whether MRP14 depletion affects accumulation of MRP8^+^ cells, the density of MRP8^+^ cells were compared between WT and MRP14KO mice during *L*. *major* or *L*. *donovani* infection. In *L*. *major* infection, although accumulation of MRP8+ cells was induced in MRP14KO mice, the degree of the cell accumulation was lower in the KO mice compared with WT mice (Fig 3A). Accumulation of MRP8^+^ cells was also found in the spleen of *L*. *donovani*-infected MRP14KO mice (Fig 3B). In contrast to the case of skin lesion in *L*. *major* infection, the degree of the cell accumulation was comparable between WT and KO mice during *L*. *donovani* infection (Fig 3C). Means ± SD of the number of MRP8^+^ cells in the red pulp of the spleen in naive and 24 week-infected WT mice were 628 ± 56 cells/mm^2^ and 1,467 ± 145 cells/mm^2^, and those in MRP14KO mice were 516 ± 106 cells/mm^2^ and 1,400 ± 181 cells/mm^2^ (Fig 3C).

### No apparent influence of MRP14 depletion on antigen-specific T cell responses during *Leishmania* infection

**Fig 4:**
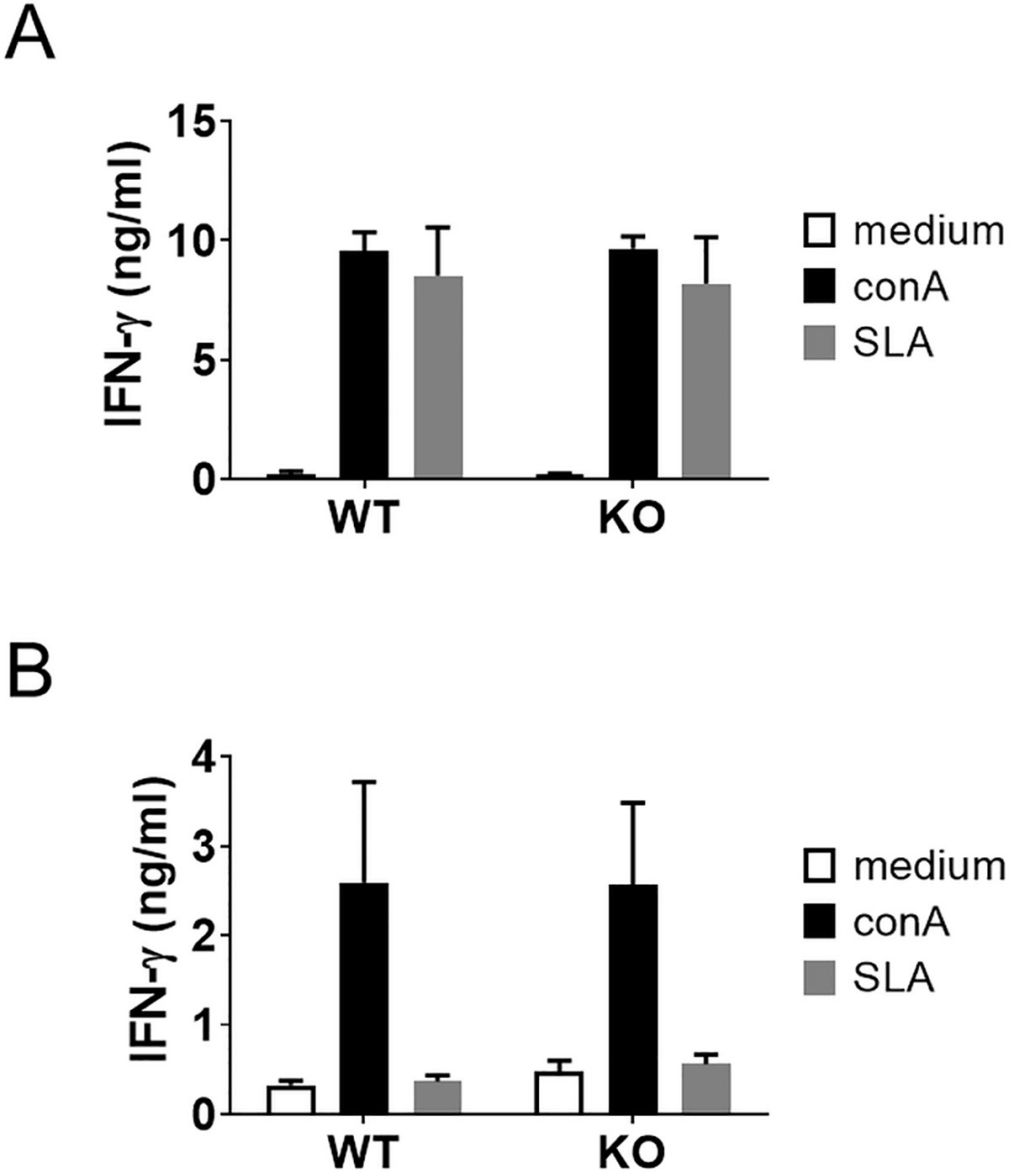
Little influence of MRP14 depletion on antigen-specific T cell responses during *Leishmania* infection. WT (n = 5) and MRP14KO mice (n = 5) were infected with *L*. *major* (A) or *L*. *donovani* (B). Splenocyte from the infected mice were either left unstimulated or stimulated with con A or SLA in vitro. After 72 hours, the supernatants were collected and measured for IFN-γ levels by sandwich ELISA Mean and SD of each group are shown. These are a representative of two or three independent experiments with similar results.

To examine if the absence of MRP14 affects the induction of T cell responses during *Leishmania* infection, splenocytes were harvested from mice infected with *L*. *major* or *L*. *donovani* and stimulated in vitro with SLA for 3 days. IFN-γ secretion from splenocytes was induced by recall with SLA and there was no difference between WT and MRP14KO mice with *L*. *major* infection (Fig 4A). In contrast, antigen-specific T cell reactivity was minimal during *L*. *donovani* infection. Splenocytes from *L*. *donovani*-infected mice did not produce much IFN-γ level upon recall with SLA, and the responses were not significantly different between WT and MRP14KO mice (Fig 4B).

### Up-regulated expression of NOSs and IFN-γ mRNA in the spleen of MRP14KO mice during *L*. *donovani* infection

**Fig 5:**
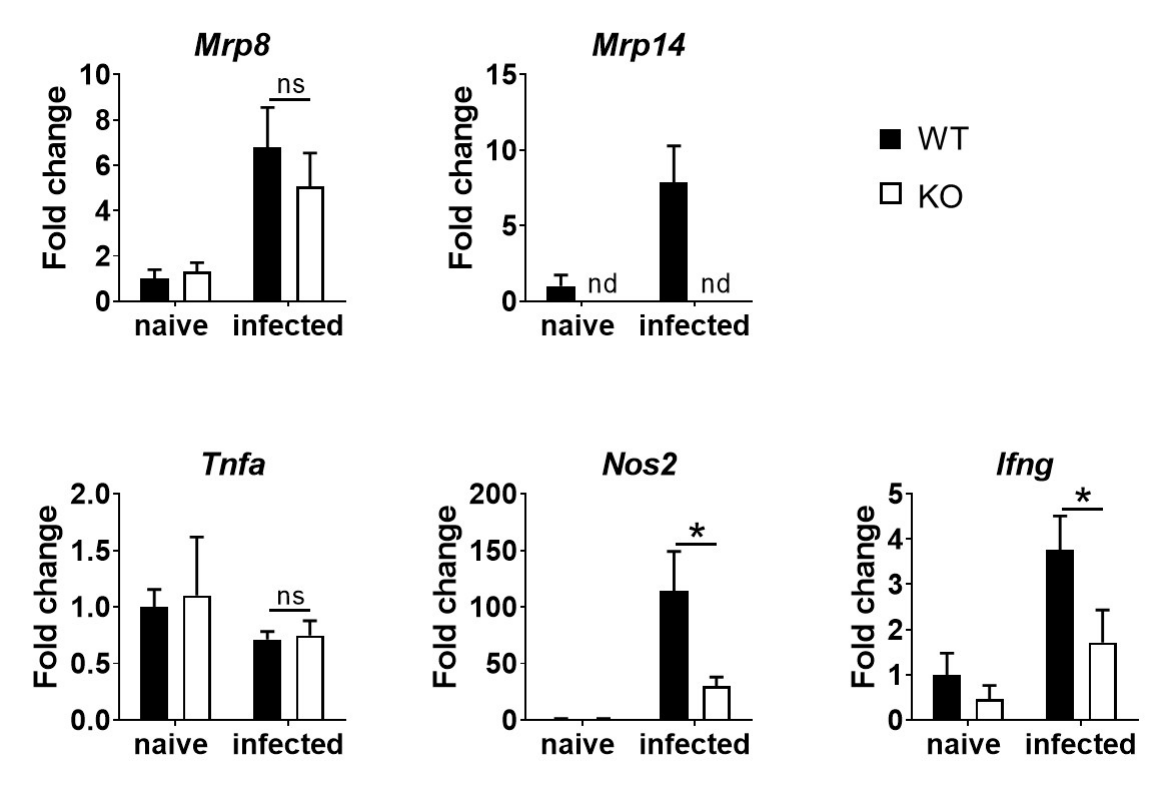
MRP14-dependent expressions of IFN-γ and NOS2 genes in the spleen of *L*. *donovani*-infected mice. The spleens were harvested from naïve or *L*. *donovani*-infected WT (n = 4 or 5) and MRP14KO mice (n = 4 or 5) and total RNA was purified from the tissues. Expression levels of MRP8, MRP14, TNF-α, NOS2 and IFN-γ mRNA in the organs were measured by quantitative PCR. Mean and SD of each group are shown. ns, not significant, *P < 0.05 by Mann-Whitney test. These are a representative of three independent experiments with similar results.

Because anemia and splenomegaly were alleviated in MRP14KO mice than WT mice during *L*. *donovani*, inflammatory responses in the spleen of *L*. *donovani*-infected mice were analyzed by measuring mRNA levels of inflammation-associated proteins. Although the mRNA expression of MRP8 and MRP14 were up-regulated after 24-week infection in WT mice, the level of MRP8 was similarly up-regulated in infected MRP14KO mice (Fig 5). The expression level of tumor necrosis factor-α was unchanged at 24 weeks post infection in both WT and MRP14KO mice (Fig 5). In contrast, NOS2 and IFN-γ mRNA levels in WT mice at 24 weeks post infection were remarkably up-regulated than in naïve mice, while the levels in MRP14KO mice were significantly lower compared to WT mice (Fig 5).

## Discussion

MRP14 is associated with inflammatory diseases [38]. In the present study, pathological roles of MRP14 in *L*. *major* and *L*. *donovani* infection were examined by using MRP14KO BALB/c mice we have previously generated [29]. Results in this study demonstrated different involvement of MRP14 in pathology during the two infections.

A previous study shows a protective role of MRP14 during *L*. *major* infection by using MRP14KO C57BL/6 mice [28]. Because the roles of MRP14 in inflammatory responses may differ in mice with different genetic backgrounds [29], we first evaluated the protective role of MRP14 in infection of BALB/c mice with *L*. *major*. In the ear infected with *L*. *major*, accumulation of MRP14^+^ cells and MRP8^+^ cells were induced (Fig 1), and the accumulation seemed to be dependent in part on endogenous MRP14 (Fig 3). It is reported that transfer of MRP8^+^ and MRP14^+^ cell induces further accumulation of MRP8^+^ and MRP14^+^ cells during *L*. *major* infection [39]. This report is in concordance with the presented result. MRP8/MRP14 are known to induce the production of proinflammatory chemokines by microvascular endothelial cells [40], accumulation of MRP8^+^ and MRP14^+^ cells to the infection site followed by secretion of those molecules may induce a positive feedback on the inflammatory response in *L*. *major* infection. MRP14 depletion exacerbated skin lesion development and ulcer formation in *L*. *major* infection without affecting the parasite burden (Fig 2). Contreras *et al*. demonstrated that injection of recombinant MRP14 into *L*. *major* infection site relieves lesion development and also reduces parasite burden, and treatment with anti-MRP8/14 does the opposite [28]. Actually, they also showed by in vitro experiments that MRP8 and MRP14 can activate macrophages to kill parasites with up-regulated TNF and iNOS [28]. In our study, parasites burdens in the lymph node were not statistically different between WT mice and MRP14KO mice with *L*. *major* infection. Therefore, it is possible that although MRP14 has a role in parasite clearance during *L*. *major* infection, control of lesion development by MRP14 is operated by other mechanisms. But how the loss of MRP14 or the resulting suppressed accumulation of MRP8^+^ cells affect the lesion worsening is still elusive and further studies are needed. We did not measure parasite burdens in the ear in this study because parasite burdens in the ear and the draining lymph node are generally proportional [41], but it is possible that MRP14 has a protective role not in lymph nodes but in the ear. This should be also addressed in future studies.

During *L*. *donovani* infection, accumulation of MRP14^+^ cells and MRP8^+^ cells was observed in both spleen and liver (Fig 1). Although the cell accumulation into the infection sites is common between CL and VL, contribution of MRP14 to MRP8^+^ cell accumulation was different between those models. The degree of MRP8^+^ cell accumulation to the spleen in MRP14KO mice was comparable that in WT mice either during *L*. *donovani* infection, whereas the cell accumulation was suppressed in the KO mice during *L*. *major* infection (Fig 3). These results suggest that MRP14 can act differently in inflammatory responses on distinct infections. Intriguingly, MRP14 depletion resulted in improvement of anemia and splenomegaly but not for hepatomegaly (Fig 2).

So, how does MRP14 act differently on pathology of leishmaniasis? Control of skin lesion in CL and exacerbation of anemia in VL, which sound opposite, are both regulated by MRP14. One explanation can be variable roles of MRP8^+^ and MRP14^+^ cells as host cells for *Leishmania* parasites. It is reported that MRP14^+^ cells have different characteristics from F4/80^+^ cells in terms of infectivity and parasite clearance [39]. We previously reported that MRP8^+^ and MRP14^+^ cells rather than F4/80^+^ cells are the major subtype of macrophages in the skin lesion during *L*. *major* infection [27]. A similar result has been reported in *L*. *amazonensis* infection; CD11b^+^ cells but not F4/80^+^ cells are dominant infected cells in the infected footpad [42]. In contrast, infected macrophages in the spleen are F4/80^+^ [43], but not MRP14^+^ or MRP8^+^ during *L*. *donovani* infection (Fig 1).

If characters of the host macrophages are different between *L*. *major* and *L*. *donovani* infection, it is possible for MRP14 to act differently on parasite clearance and pathology during CL and VL. For example, such differences can be derived from different repertoires of MRP14 receptors on host macrophages. Multiple receptors including TLR2, TLR4 and RAGE are proposed as MRP14 receptors and the function of MRP14 varies depending on which receptor it binds. MRP14 induces inflammatory cytokine production via TLR4, while MRP14 induces leukocyte migration such as granulocytes, monocytes and lymphocytes via RAGE [15]. Besides, MRP14 induces accumulation of myeloid derived suppressor cells via RAGE [44]. Although outcomes of MRP14 signaling via TLR2 are still unknown, it is possible that each receptor has a different role and the expression pattern of the receptors at the infection sites changes the role of MRP14. Therefore, further characterizations on MRP14^+^ cells in terms of the receptor expressions are needed to understand the different outcomes of MRP14 depletion between CL and VL.

Or, the same effector molecules downstream of MRP14 can have bilateral effects on pathology during leishmaniasis, i.e., protectively in CL and detrimentally in VL. Quantitative PCR analyses demonstrated that MRP14 acts as an enhancer for NOS2 and IFN-γ mRNA expressions in the spleen of *L*. *donovani*-infected mice (Fig 5). It is well known that these two molecules contribute to control of CL [45,46], whereas they may promote anemia during VL. We previously reported that infected macrophages in the spleen engulf erythrocytes during VL and proposed that the phenomenon, called hemophagocytosis, is one of the possible causes of anemia during VL [43]. Hemophagocytosis results from hyper-activation of macrophages by many reasons. In the case of VL, most of the hemophagocytes in the spleen were multinucleated giant cells (MGC) heavily infected with *L*. *donovani* [43]. IFN-γ has a crucial role in MGC formation [47]. Interestingly, Zoller *et al*. reported that systemic exposure to physiologically relevant levels of IFN-γ is sufficient to cause hemophagocytosis [48]. In addition, IFN-γ-deficient mice do not exhibit hemophagocytosis and anemia during *Trypanosoma brucei* infection [49]. These reports suggest that MRP14 contribute to development of anemia by inducing IFN-γ which results in up-regulated hemophagocytosis, at least in the status that infection is not well controlled. But, the major source of IFN-γ during *L*. *donovani* infection may not be T cells since antigen-specific induction of IFN-γ production was not observed in splenocytes from the infected MRP14KO mice (Fig 4) NK cells and ILC1 cells are known innate immune cells as a source of IFN-γ [50]. MRP14 can activate NK cells and support IFN-γ production [51,52], suggesting the molecule affects immune responses through direct activation of innate immunity rather than manipulating acquired immunity.

In conclusion, we have demonstrated the different pathological roles of MRP14 during experimental CL and VL. To our knowledge, this is the first report addressing the roles of MRP14 in *L*. *donovani* infection. Although MRP14 is associated with a wide range of inflammatory diseases, the functions remain elusive. Our study that the functions vary even between *L*. *major* and *L*. *donovani* infection may prove the complexity of MRP14, but at the same time support understanding of the mechanisms behind the complexity. Nonetheless, studies using MRP14-KO mice are insufficient to elucidate the complex roles of MRP14 in infectious diseases, and further studies using MRP14-reporter mice, MRP14-conditional KO mice, MRP14 inhibitors and anti-MRP14 antibody for flow cytometry are definitely required. Development of such the research tools will lead to understanding of multifunctional roles of MRP14.

## Supporting information

S1 Table Primer list for quantitative PCR analyses. (DOCX)
