# Supplementary material for: Pathological roles of MRP14 in anemia and splenomegaly during experimental visceral leishmaniasis
Source: PLoS Negl Trop Dis. 2020 Jan 21;14(1):e0008020. doi: 10.1371/journal.pntd.0008020 (PMC6994150; doi:10.1371/journal.pntd.0008020)
Supplement: S1 Table — (DOCX) [file pntd.0008020.s001.docx]

Supplementary Table 1. Primer list for quantitative PCR analyses

| Gene |  | Primer sequence |
| --- | --- | --- |
| *Gapdh* | F | 5'-CGACTTCAACAGCAACTCCCACTCTTCC-3' |
|  | R | 5-TGGGTGGTCCAGGGTTTCTTACTCCTT-3′ |
| *Mrp8* | F | 5'-GGAAATCACCATGCCCTCTA-3' |
|  | R | 5'-TGGCTGTCTTTGTGAGATGC-3' |
| *Mrp14* | F | 5'-CAGCATAACCACCATCATCG-3' |
|  | R | 5'-GTCCTGGTTTGTGTCCAGGT-3' |
| *Tnfa* | F | 5'-CTGTGAAGGGAATGGGTGTT-3' |
|  | R | 5'-GGTCACTGTCCCAGCATCTT-3' |
| *Nos2* | F | 5'-GTTCTCAGCCCAACAATACAAGA-3' |
|  | R | 5'-GTGGACGGGTCGATGTCAC-3' |
| *Ifng* | F | 5'-GGCCATCAGCAACAACATAAGCG-3' |
|  | R | 5'-TGGGTTGTTGACCTCAAACTTGG-3' |
